# Improving item pool utilization for health professions examinations under variable-length computerized adaptive testing designs: a shadow-test approach

**DOI:** 10.3352/jeehp.2025.22.35

**Published:** 2025-11-03

**Authors:** Hwanggyu Lim, Kyung (Chris) Tyek Han

**Affiliations:** 1 College of Education, Inha University, Incheon, Korea; 2 Graduate Management Admission Council, Reston, VA, USA; Hallym University, Korea

**Keywords:** Algorithms, Computer simulation, Computerized adaptive testing, Health occupations, Medical licensure, Republic of Korea, United States

## Abstract

**Purpose:**

The shadow-test approach to computerized adaptive testing (CAT) ensures content validity in health professions examinations but may suffer from poor item pool utilization in variable-length designs, increasing operational costs and security risks. This study aimed to address this challenge by developing algorithms that enhance the sustainability of shadow CAT in variable-length design.

**Methods:**

A simulation study was conducted to evaluate 3 proposed modifications of the α-stratification method designed to improve item pool utilization. These methods, which integrated randomesque selection and multiple-form strategies, were compared with 2 baseline algorithms within a variable-length shadow CAT framework. Performance was assessed in terms of measurement precision, pool utilization, and test efficiency.

**Results:**

The proposed modifications significantly outperformed the baseline methods across all measures of item pool utilization and exposure control. The most effective method (Modification 2) reduced the proportion of unused items from 35.6% to 5.0% and produced more uniform item exposure rates. These substantial gains in operational sustainability were achieved while maintaining measurement precision comparable to the baseline methods.

**Conclusion:**

The proposed algorithms effectively mitigate poor item pool utilization in shadow CAT under variable-length design. This enhanced framework provides a robust, secure, and sustainable solution for high-stakes adaptive assessments in the health professions that remain content-valid, precise, and operationally efficient.

## Graphical abstract

[Fig f4-jeehp-22-35]

## Introduction

### Background

High-stakes examinations for health professionals, such as medical and nursing licensure tests, serve a critical gatekeeping function to ensure that candidates possess the competence required for safe and effective patient care. The validity and fairness of these assessments are therefore paramount. Accordingly, many credentialing bodies are increasingly transitioning from traditional paper-and-pencil formats to computerized adaptive testing (CAT). This shift, evident in examinations such as the National Council Licensure Examination (NCLEX) and under consideration for the Korean Medical Licensing Examination (KMLE), is driven by the significant advantages of CAT, including enhanced measurement precision, reduced testing time, and improved test security.

Despite these advantages, the sequential item-selection process of conventional CAT introduces a crucial trade-off between measurement precision and content validity. While the algorithm maximizes information at each step, it cannot guarantee that the final set of administered items adheres to the complex content blueprints essential for a valid assessment. This challenge is particularly acute in health professions examinations, which must encompass diverse clinical domains, cognitive levels, and patient scenarios. Inconsistent content representation across candidates thus poses a direct threat to the validity of assessment outcomes [1]. Although several heuristic methods have been developed to manage content balancing (e.g., maximum priority index [2], normalized weighted absolute deviation heuristic [3], weighted penalty model [4], weighted deviation method [5]), these approaches rely on weights and penalties and therefore cannot ensure strict compliance with all test constraints, particularly when the blueprint is complex [6,7].

The shadow-test approach (STA) addresses this trade-off by integrating optimal test assembly principles directly into the adaptive process, a procedure known as shadow CAT [6,8-10]. The STA reconceptualizes CAT as a sequence of real-time test assembly problems in which, following each response, a full-length shadow test is assembled. This shadow test is constructed using mixed-integer programming (MIP) to be optimal for the candidate’s current ability estimate while simultaneously satisfying all content and statistical constraints [9]. The next item administered is then selected from this optimal, content-valid shadow test. This forward-looking process ensures that a feasible, blueprint-compliant path to test completion always exists, thereby eliminating the risk of constraint violations [11]. These properties make the STA particularly suitable for the complex requirements of high-stakes assessments in the health professions [6].

CAT designs are primarily distinguished by their stopping rules: fixed-length or variable-length [12]. While fixed-length tests administer a constant number of items, variable-length tests continue until a specific level of measurement precision, typically a target standard error (SE) of the ability estimate, is achieved. For health professions credentialing exams such as the NCLEX, which make critical pass/fail classifications based on a cut-score, the variable-length design is particularly advantageous. This approach improves testing efficiency by tailoring the test length to each candidate, ensuring that all classification decisions are supported by sufficient statistical evidence [13].

Despite the theoretical superiority of the STA, its application in variable-length CAT designs reveals a significant operational limitation: poor item pool utilization. For example, in a large-scale simulation study, Diao and Ren [8] found that while shadow CAT in variable-length outperformed heuristic methods in precision and constraint satisfaction, nearly 20% of the item bank was never administered. This inefficiency leads to overexposure of a small subset of items and underuse of the majority, which in turn increases test security risks and inflates long-term item development costs. For large-scale testing programs, such as those in the health professions, this poor pool utilization presents a critical drawback, necessitating the development of more robust item selection algorithms that can effectively manage item exposure while preserving the core benefits of the shadow CAT framework.

### Objectives

This study therefore aims to address the critical challenge of poor item pool utilization in shadow CAT under variable-length design. To this end, we propose and evaluate 3 modifications of the α-stratification (ASTR) item selection method [14]. The objective is to establish a practical methodology that enhances item pool utilization and exposure control, thereby ensuring that adaptive assessments for the health professions are not only precise and content-valid but also operationally sustainable and secure for long-term administration.

## Methods

### Ethics statement

This study was based on a computer simulation; therefore, no institutional review board approval or informed consent was required.

### Study design

This study employed a simulation-based design to evaluate 5 item selection algorithms—including 3 newly proposed modifications—within a variable-length shadow CAT framework. The simulation was modeled to reflect a high-stakes credentialing examination, such as those used in the health professions. The fundamental procedure of shadow CAT [9] and the specific 2-stage design for variable-length tests [8] used in this study are described below.

#### The Shadow CAT Procedure

**Fig. 1.**
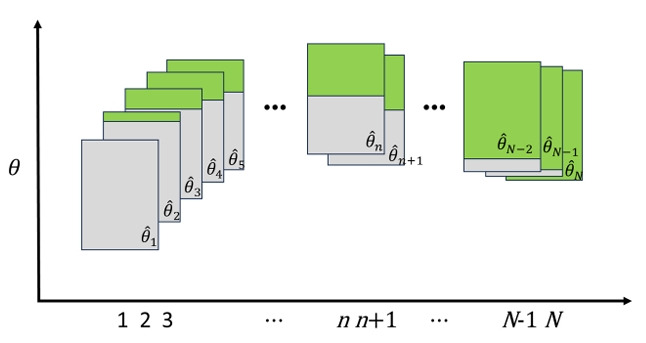
Graphical illustration of the shadow computerized adaptive testing (CAT) framework. The vertical axis indicates the ability (*θ*) and the horizontal axis denotes the item administration order during the CAT.

The shadow CAT framework operates as an iterative process, as graphically illustrated in Fig. 1. In this framework, each rectangular block in the figure represents a potential shadow test at a given step of the iterative assembly process, formulated and solved using MIP. The green portion of each shadow test block indicates items that have already been administered to the examinee in previous steps, whereas the gray portion represents items currently available in the shadow test but not yet administered. To select the (n+1)th item for an examinee who has already completed n items, the following steps are executed in real time:

Ability estimation: First, the examinee’s ability (${\hat{\theta }}_{n}$) is estimated based on their responses to the n items already administered.

Shadow test assembly: Next, using the updated ${\hat{\theta }}_{n}$ as the optimization target, a full-length shadow test is then assembled from the available item pool using MIP. Critically, the MIP model is constrained to include the n items already administered. The solver selects the remaining items to construct a complete shadow test that maximizes statistical information at ${\hat{\theta }}_{n}$ while simultaneously satisfying all other pre-specified test constraints (e.g., content balance).

Item selection and administration: The (*n*+1)th item is then selected from the set of unadministered items within this newly assembled, optimal, and content-valid shadow test. Typically, the item with the maximum Fisher information (MFI) at ${\hat{\theta }}_{n}$ is chosen and administered. This cycle repeats until a test termination rule is met.

#### The 2-stage shadow CAT in variable-length

This study implemented the general shadow CAT framework using the 2-stage variable-length procedure proposed by Diao and Ren [8].

Stage 1 (minimum requirements phase): The test continues until a predefined minimum number of items has been administered (e.g., 32 items in this study). The goal of this stage is to ensure that each test meets the minimum core content specifications required for a valid assessment. During this stage, each assembled shadow test is constrained by the minimum test length.

Stage 2 (precision-targeting phase): Once the minimum length is reached, the assembly process is reconfigured to use the maximum allowable test length as the new constraint (e.g., 48 items in this study). After each item is administered, the test terminates when either the target SE of the ability estimate is achieved or the maximum test length is reached.

### Item selection algorithms and proposed modifications

To address the issue of poor item pool utilization, this study proposed and evaluated 3 modifications to the standard ASTR approach [14]. These 3 modified algorithms were compared against 2 baseline methods: the standard MFI-only algorithm and the ASTR-only algorithm.

The MFI-only method serves as the simplest baseline, selecting the single most informative unadministered item from the shadow test at each step. The ASTR-only method replicates the approach used by Diao and Ren [8], in which the item pool is partitioned into 3 strata based on a-parameter values, and the most informative items are selected sequentially from the lowest to the highest stratum.

Building on the ASTR framework, the following 3 modifications were proposed, all of which integrate the randomesque item selection strategy [15]:

#### Modification 1 (α-stratification with randomesque)

This approach enhances the standard ASTR method by incorporating a random selection component based on the randomesque method [15]. While the ASTR-only method is deterministic, always selecting the single most informative item from the eligible stratum, Modification 1 introduces a probabilistic step. At each selection point, a small set of the most informative items (candidates) is first identified within the shadow test, and the next item is randomly chosen from this set. By preventing the algorithm from repeatedly selecting the same single best item, this added randomness is hypothesized to promote more uniform item exposure and improve overall item pool utilization. For this study, the size of the candidate set decreased as the algorithm progressed through the a-strata (4 in the lowest stratum, 3 in the middle, and 2 in the highest) during Stage 1 and was fixed at 2 during Stage 2.

#### Modification 2 (parallel shadow forms)

This method extends Modification 1 by incorporating the use of multiple shadow tests, a concept originally suggested by van der Linden [9] to reduce item exposure rates in fixed-length CAT. At each selection step, 2 parallel shadow tests are constructed simultaneously under the critical constraint that they cannot share any unadministered items. This “no-item overlap” constraint forces the MIP solver to identify 2 disjoint sets of optimal items, thereby diversifying the high-quality candidate items available at each step. This diversification is expected to improve item pool utilization and exposure control. The trade-off is a potential reduction in efficiency, as one form may include items with slightly lower information than would otherwise be selected without the overlap constraint.

#### Modification 3 (multiple forms with ability uncertainty)

Similar to Modification 2, this approach also constructs multiple shadow tests simultaneously but explicitly accounts for uncertainty in $\hat{\theta }$. Three shadow tests are assembled: one optimized at the current $\hat{\theta }$, one at $\hat{\theta }-SE/2$, and one at $\hat{\theta }+SE/2$. Unlike Modification 2, overlap of unadministered items between these forms is permitted. This design incorporates estimation uncertainty directly into the item selection process. By allowing overlap, this method may balance the increased candidate set size of a multi-form approach with the psychometric optimality of a single-form approach, as all forms can still select the most informative items if necessary. As the test progresses and SE decreases, the 3 optimization points converge, leading to a greater degree of item overlap among the multiple shadow tests during the later stages of the CAT.

### Participants

Two distinct samples of simulated examinees were generated. The first sample (overall sample), used to assess overall performance, consisted of 50,000 examinee abilities (*θ*s) drawn from *N*(0, 1). The second sample (conditional samples), used for conditional analyses, consisted of 6,500 examinees, with 500 examinees simulated at each of 13 discrete ability levels ranging from –3.0 to 3.0 in increments of 0.5. These sample sizes were chosen to ensure stable and reliable estimation of the evaluation criteria. For all simulations, the initial ability estimate was set to $\hat{\theta }$=0.

### Variables

The primary independent variable was the item selection algorithm, with 5 levels: (1) MFI-only, (2) ASTR-only, (3) Modification 1, (4) Modification 2, and (5) Modification 3. The dependent variables were a set of evaluation criteria organized into 3 categories:

#### Measurement precision

Bias, root mean squared error (RMSE), and the average SE of the final $\hat{\theta }$.

#### Pool utilization and exposure control

The proportion of over-exposed (exposure rate >0.3) and under-exposed (exposure rate <0.02) items, the proportion of unused items, the chi-square (χ^2^) index for uniformity of exposure rates [14] and the test overlap rate [14].

#### Test efficiency

The average test length, an index of efficiency [16], and the proportion of examinees reaching the maximum test length without satisfying the SE criterion.

### Data sources/measurement

An item pool of 360 items was simulated using the 3-parameter logistic (3PL) item response theory (IRT) model. The item pool size was chosen to be comparable to that of the current fixed-form KMLE. Item parameters were generated from distributions representative of operational testing programs (e.g., [17]): discrimination parameters (a) from N(1.0, 0.4), difficulty parameters (b) from N(0.0, 1.0), and guessing parameters (c) from Beta(8, 32). Each item was randomly assigned to 1 of 4 content categories, with each category containing 90 items (25% of the pool).

The CAT was configured with a minimum test length of 32 items (Stage 1) and a maximum of 48 items (Stage 2). The termination rule for Stage 2 was a target SE of 0.2. The test blueprint required that the final test include a minimum of 8 and a maximum of 12 items from each of the 4 content categories. For the ASTR and 3 modified algorithms, the item pool was divided into 3 equal-sized strata based on a-parameter values. During Stage 1, the first 14 items were selected from the lowest a-stratum and the next 12 from the middle a-stratum. The remaining 6 items in Stage 1, as well as all items in Stage 2, were selected from the entire pool. The randomesque candidate set size for the 3 modified algorithms was set to 4 for the first stratum, 3 for the second, and 2 for all subsequent items.

### Statistical methods

Interim ability estimates were updated using weighted maximum likelihood estimation (MLE) [18]. The final ability estimate was calculated using MLE with bounds set at [–4, 4].

To assess measurement precision, bias and RMSE were computed by comparing the final $\hat{\theta }$ to the true *θ* for each of *m* examinees:


$$Bias=\;\frac{\sum_{i=1}^{m}({\hat{\theta }}_{i}-\theta_{i})}{m},$$

$$RMSE=\;\sqrt{\frac{\sum_{i=1}^{m}{\left({\hat{\theta }}_{i}-\theta_{i}\right)}^{2}}{m}.}$$

For pool utilization and exposure control, the χ² index and the test overlap rate were computed [14]. The χ² index measures the deviation of observed item exposure rates (*er_j_*) from the ideal uniform exposure rate (*L/N*), where L is the average test length and *N* is the item pool size:


$$\chi^{2}=\;\overset{N}{\underset{j=1}{\sum }}\frac{{\left(er_{j}-\frac{L}{N}\right)}^{2}}{\frac{L}{N}}.$$

The test overlap rate was defined as the average proportion of common items between pairs of randomly selected examinees [14].

Finally, test efficiency was quantified using an index proposed by Huo [16], which represents the average test information accumulated per administered item across all examinees:


$$Efficiency=\frac{\sum_{i=1}^{m}TI_{i}}{\sum_{i=1}^{m}L_{i}}$$

where *TI_i_* is the test information accumulated for examinee *i* over the course of the test.

All simulations and analyses were performed in R [19]. The MIP component for shadow-test assembly was implemented using lp_solve 5.5 [20] via the lpSolveAPI package [21]. Key IRT procedures, including the generation of simulated response data, computation of item information, and ability estimation, were conducted using the irtQ package [22]. Illustrative R scripts, including source code and item pool file, are provided in the supplementary materials (Supplements 1, 2).

## Results

### Overall sample results

Table 1 presents the measurement precision results for the overall sample. All 5 item selection methods produced comparable and negligible bias. Although the MFI-only method yielded a slightly lower RMSE, the absolute differences among the 5 methods were minimal (maximum RMSE difference=0.007). Notably, the average final SE for the MFI-only method was slightly below the 0.2 termination criterion, whereas the averages for the other 4 methods were slightly above it. Overall, these findings indicate that the proposed modifications maintained a high level of measurement precision comparable to the baseline approaches.

As shown in Table 2, the 3 proposed modifications demonstrated markedly superior performance in item pool utilization and exposure control compared with the baseline methods. Modification 2 was the most effective, reducing the percentage of unused items to just 5.0%, in sharp contrast to 35.6% for the MFI-only method and 20.3% for the ASTR-only method. Modification 2 also achieved the most uniform item exposure, evidenced by the lowest χ² index (34.338) and the lowest test overlap rate (21.4%). Among the other 2 modified approaches, Modification 3 showed a slight advantage over Modification 1 in these metrics.

Test efficiency results, summarized in Table 3, revealed the expected trade-off between pool utilization and test length. The MFI-only method produced the shortest average test length (33.7 items). In contrast, the proposed modifications required slightly longer tests (41.2 to 42.5 items) to achieve their gains in pool utilization and test security. Nevertheless, their efficiency levels were comparable to those of the ASTR-only method, suggesting that substantial operational improvements were obtained at a modest cost. Although Modification 2 had the highest proportion of examinees who did not reach the target SE, this difference was only marginal compared with the ASTR-only and other modified methods.

### Conditional sample results

**Fig. 2.**
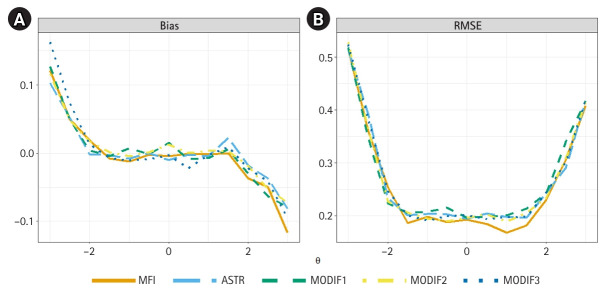
Conditional bias (A) and root mean squared error (RMSE) (B) of final ability estimates for the 5 item selection algorithms across the ability scale. MFI, maximum Fisher information only; ASTR, α-stratification only; MODIF1, Modification 1; MODIF2, Modification 2; MODIF3, Modification 3.

Fig. 2 presents the conditional bias and RMSE for the 5 methods across the *θ* scale. For examinees within the central ability range (*θ*=–1.5 to 1.5), all 5 approaches demonstrated comparable measurement precision, with conditional biases near zero. Within this same range, the conditional RMSEs for the ASTR-only method and the 3 proposed modifications hovered around the target SE of 0.2, whereas the MFI-only method produced slightly lower RMSEs.

**Fig. 3.**
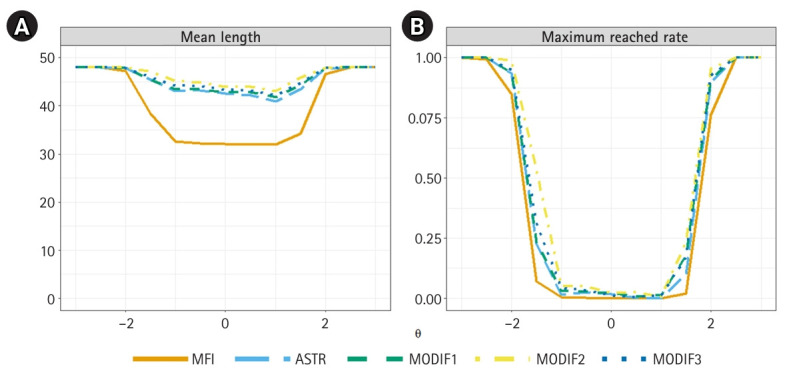
Conditional test efficiency for the 5 item selection algorithms, showing the average test length (A) and the proportion of examinees reaching the maximum test length (B) across the ability scale. MFI, maximum Fisher information only; ASTR, α-stratification only; MODIF1, Modification 1; MODIF2, Modification 2; MODIF3, Modification 3.

Fig. 3 illustrates the conditional test efficiency of the 5 methods. As shown in the left panel, the MFI-only method consistently yielded the shortest average test lengths for examinees in the middle of the *θ* range. In contrast, the other 4 methods—designed to improve pool utilization—required somewhat longer tests in this central region but exhibited similar efficiency patterns to one another. For examinees at the extremes of the *θ* scale (*θ*<–2.0 and *θ*>2.0), this efficiency difference disappeared, as the average test length for all 5 approaches converged toward the maximum of 48 items. The right panel further clarifies this outcome, showing that the rate of reaching the maximum test length approached 100% for all methods at these ability extremes, reflecting the limited information available in the item pool to meet the target SE criterion.

## Discussion

### Key results

This study aimed to address the problem of poor item pool utilization in shadow CAT under variable-length design—a critical barrier to its implementation in high-stakes testing. The simulation results demonstrate that the 3 proposed modifications to the ASTR method successfully achieved this goal. All 3 modifications substantially outperformed the baseline MFI-only and ASTR-only methods in terms of item pool utilization and exposure control, while maintaining a comparably high level of measurement precision. In particular, Modification 2, which utilized parallel shadow forms without item overlap, proved most effective, reducing the rate of unused items from over 35.6% in the MFI-only method to just 5.0%.

### Interpretation

The superior performance of the proposed methods can be attributed to the integration of controlled randomness into the item selection process. The MFI-only and ASTR-only methods, being more deterministic, tend to repeatedly select from a small subset of highly informative items, leaving much of the pool underused. The introduction of the randomesque component—especially when amplified by the larger candidate sets in Modifications 2 and 3—effectively diversified item selection. This broader and more uniform sampling of items significantly enhances test security and the long-term sustainability of the item bank.

The performance differences among the modified methods are also instructive. The success of Modifications 2 and 3 stems from their use of multiple shadow tests to generate a larger and more varied set of candidate items at each step. Modification 2, however, yielded the best results because the no-item-overlap constraint forced the test assembler to explore completely distinct sets of items, thereby maximizing diversity among candidate sets. In contrast, although Modification 3 effectively incorporated ability uncertainty, allowing item overlap meant that its candidate sets could be more similar, slightly diminishing its impact on pool utilization relative to Modification 2.

The observed improvement in pool utilization came with a modest and expected cost to test efficiency, reflected in a slight increase in average test length compared with the MFI-only method. This outcome highlights the inherent trade-off in CAT between psychometric efficiency and operational constraints such as exposure control. Importantly, however, the efficiency of the modified methods remained comparable to that of the ASTR-only method, indicating that substantial gains in pool utilization and test security were achieved without a significant additional loss in efficiency. These findings suggest that the proposed algorithms provide a balanced and sustainable solution, optimizing pool utilization without compromising measurement precision or efficiency.

### Comparison with previous studies

The findings of this study directly address and resolve the key drawback of the variable-length shadow CAT framework identified by Diao and Ren [8]. While their work confirmed the superiority of shadow CAT in satisfying test constraints, its poor pool utilization can be a major operational limitation. The current study demonstrates that this weakness is not inherent to the shadow CAT framework itself but can be effectively mitigated through algorithmic enhancements. The use of parallel forms (Modification 2) and multiple forms accounting for ability uncertainty (Modification 3) extends the work of van der Linden [9] by illustrating how these principles can be successfully adapted to improve pool utilization in a variable-length context.

### Limitations

This study has several limitations. First, the findings are based on a simulation study. Although the simulated item pool and testing conditions were designed to be realistic, actual performance in an operational setting with a live item pool and examinees may differ. Second, the effectiveness of the proposed modifications, particularly those involving multiple shadow tests, depends on the availability of a sufficiently large and high-quality item pool to ensure that feasible solutions can always be generated. Further research is needed to evaluate the performance of these methods with smaller or more heavily constrained item banks. Third, the simulation did not model the positive correlation often observed between item discrimination and difficulty parameters in practice [23,24], which may influence the effectiveness of ASTR.

### Generalizability and suggestions

Despite these limitations, the findings hold strong practical relevance for high-stakes credentialing programs in the health professions. For testing programs considering a transition to CAT, such as the NCLEX or the KMLE, this study provides a refined shadow-test framework for implementing a variable-length design that is both psychometrically robust and operationally sustainable. The use of a 360-item pool in the simulation, mirroring the current fixed-form KMLE, further enhances its practical applicability. These results demonstrate that the proposed modifications to the shadow CAT framework can be effectively implemented within the constraints of existing or modestly sized item pools, enabling testing programs to adopt advanced adaptive testing models without the immediate need for an excessively large item pool. This, in turn, can reduce item development costs and facilitate smoother transitions to adaptive testing.

### Conclusion

Although variable-length shadow CAT offers unparalleled control over content validity in health professions examinations, its operational use has been hindered by poor item pool utilization. This study addressed this limitation by introducing and validating 3 modified ASTR algorithms that substantially improve item pool usage and exposure control while maintaining high measurement precision. The findings establish the enhanced framework as a more robust, secure, and sustainable solution for the next generation of adaptive tests, ensuring fairness, validity, test security, and cost-effectiveness in high-stakes assessment environments.

## Figures and Tables

**Figure f4-jeehp-22-35:**
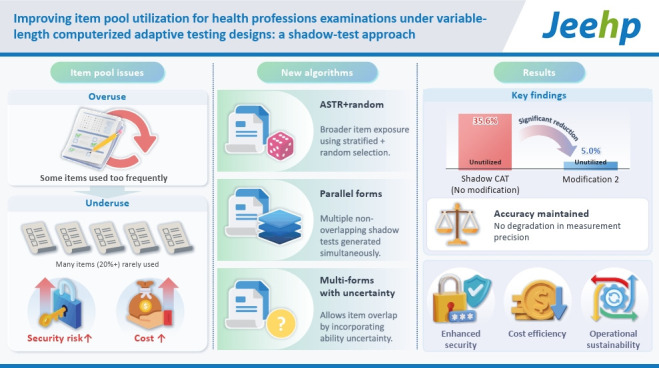


**Table 1.** Measurement precision for the overall sample

Method	Bias	RMSE	Average SE
MFI	0.007	0.204	0.196
ASTR	0.007	0.210	0.203
MODIF1	0.008	0.210	0.203
MODIF2	0.007	0.211	0.204
MODIF3	0.006	0.210	0.203

RMSE, root mean squared error; Average SE, average standard error of ability estimate; MFI, maximum Fisher information only; ASTR, α-stratification only; MODIF1, Modification 1; MODIF2, Modification 2; MODIF3, Modification 3.

**Table 2.** Item pool utilization and exposure control for the overall sample

Method	Over-exposure (%)	Less-exposure (%)	Unused items (%)	χ^2^	Test overlap (%)
MFI	10.3	53.9	35.6	87.059	33.6
ASTR	12.5	41.4	20.3	70.776	31.1
MODIF1	12.5	40.8	19.2	67.857	30.3
MODIF2	7.2	25.0	5.0	34.338	21.4
MODIF3	11.1	38.1	18.3	54.058	26.7

χ^2^=χ^2^ index of item exposure rate. MFI, maximum Fisher information only; ASTR, α-stratification only; MODIF1, Modification 1; MODIF2, Modification 2; MODIF3, Modification 3.

**Table 3.** Test efficiency for the overall sample

Method	Average test length	Unreached SE (%)	Efficiency
MFI	33.7	7.1	0.792
ASTR	41.1	8.5	0.603
MODIF1	41.2	8.7	0.601
MODIF2	42.5	10.2	0.582
MODIF3	42.0	9.2	0.590

Unreached SE (%)=percentage of simulees whose final SE did not reach the 0.2 criterion. SE, standard error; MFI, maximum Fisher information only; ASTR, α-stratification only; MODIF1, Modification 1; MODIF2, Modification 2; MODIF3, Modification 3.

## Supplementary materials

Supplement 1. README.md file providing a comprehensive guide for replicating the simulation study.

Supplement 2. A zipped file (Supplementary_Material.zip) containing all R scripts, source codes, and data files used in the study.

Supplement 3. Audio recording of the abstract.
